# Influence of the Bearing Thermal Deformation on Nonlinear Dynamic Characteristics of an Electric Drive Helical Gear System

**DOI:** 10.3390/s21010309

**Published:** 2021-01-05

**Authors:** Xianghuan Liu, Defu Liu, Xiaolan Hu

**Affiliations:** 1 State Key Laboratory of High Performance Complex Manufacturing, Central South University, Changsha 410083, China; liudefu@csu.edu.cn (D.L.); huxiaolan@hnu.edu.cn (X.H.); 2 Zhuzhou Gear Co., Ltd., Zhuzhou 421000, China; 3 State Key Laboratory of Advanced Design and Manufacturing for Vehicle Body, Hunan University, Changsha 410082, China

**Keywords:** bearing, thermal deformation, nonlinear dynamic, electric drive, gear system

## Abstract

Based on the statics and quasi-statics analysis methods, the thermal deformation calculation model of a deep-groove ball bearing was constructed for the helical gear transmission system of a high speed electric drive, and the radial and axial bearing stiffness values of the bearing were calculated under the thermal deformation in this study. The obtained radial and axial stiffness values were introduced into the established dynamics model of helical gear system, and the influence of changed bearing stiffness, resulting from the thermal deformation, on the nonlinear dynamic characteristics of gear pair was analyzed using the Runge–Kutta method. The results show that the axial and radial deformations of bearing occur due to the increase of working speed and temperature, in which the axial stiffness of bearing is improved but the radial stiffness is reduced. The decreasing degree of axial stiffness and the increasing degree of radial stiffness decrease with the gradually increasing working rotational speed. When considering the influence of thermal deformation on the bearing stiffness, the helical gear system will have nonlinear behaviors, such as single periodic, double periodic, and chaotic motion with the change of working speed. Therefore, in order to improve the nonlinear dynamic characteristics of high speed electric drive gear systems, the influence of bearing stiffness change on the dynamic performance of a gear system should be considered in the industrial applications.

## 1. Introduction

The transmission system is a core unit of automobile and train power systems, and it plays a critical role in the dynamic performance of automobile and high speed trains [1,2]. Compared with the conventional transmission system, the transmission system of a high speed electric drive has its specific characteristics of higher rotational speed and great torque, which may increase the failure risk of gear bonding. Moreover, as there is no engine cover effect in the electric drive, the vibration and noise of gear pairs may become more prominent under the working conditions of high speed and great torque, in which the better working performance and vibration control of key components, such as gears and bearings, are required [3,4,5,6]. Therefore, it is of great significance to study the dynamics and vibration characteristics of high speed electric drive [7], determining the NVH (noise, vibration, and harshness) performance of automobiles.

The dynamic characteristics of gears and bearings under the high speed working conditions have been well studied by previous researchers [8,9]. Ouyang Tiancheng et al. [3] analyzed the lubrication and dynamic characteristics of high speed spur gears. Hu Zehua et al. [10] proposed the dynamic characteristics of a high speed surface gear rotation coupling system. Kunpeng Xu et al. [11] investigated the influence of rotation speed on the nonlinear dynamic characteristics of the angular contact ball bearing. B. Choe et al. [12] studied the dynamic characteristics of ball bearings immersed in the low-temperature liquid at different speeds and loads. S. Jain [13] and Y. Wang [14] found the sliding characteristics of angular contact ball bearings with the influence of gyroscopic moment and centrifugal force at high speed. When analyzing the nonlinear dynamic characteristics of the transmission system, the bearing was generally simplified and only the radial bearing stiffness was considered, ignoring the axial stiffness of the bearing [15,16], or the axial stiffness of the bearing was regarded as a linear spring [17,18]. However, with the development of electric drive technology towards low noise, high efficiency, high speed, and multigear, the change of bearing stiffness cannot be ignored at the high speed due to the thermal deformation. Therefore, Feng Haisheng [19] and Li Tongjie et al. [20] studied the influence of bearing stiffness on the system dynamics performance under the high speed working conditions. Kahraman et al. and Ozguven et al. [21,22] established a nonlinear dynamic model of the gearing-rotor-bearing system, aiming at considering the bearing stiffness and other factors, and studied the influence of bearing stiffness on the nonlinear characteristics of the transmission system. When analyzing the dynamic characteristics of a high speed electric drive gear transmission system, the axial and radial loads not only create a contact stress between the ball and the inner ring, or the outer ring, changing its contact angle, resulting in the bearing wear failure [23], but also the temperature rise of bearing can change the dynamic stiffness [24], the dynamic response of the gear and its amplitude [25,26], which may affect the whole transmission.

In order to improve the NVH performance of a high speed electric drive transmission system [27], this paper studied the thermal deformation of bearing under the high speed working conditions and the influence of bearing stiffness, which was changed by the thermal deformation [28,29], on the nonlinear dynamic characteristics of an electric drive helical gear transmission system. Firstly, through the statics and quasi-statics analysis of bearings, the heat generation of the deep groove ball bearing was calculated for the high speed electric drive, and the temperature rise and thermal deformation of the bearing were calculated based on the thermal resistance network method. Then, the bearing stiffness values under different thermal deformation conditions were calculated and introduced into the nonlinear dynamic model of the high speed electric drive helical gear transmission system. The dynamic transfer error bifurcated diagrams of gear pair were analyzed, and the influence of bearing thermal deformation on the nonlinear dynamic characteristics of the high speed electric drive gear system was studied. The average rotating speed of car engines is around 10,667 rpm when it is running at the maximum highway speed limit of 120 km/h, and the maximum design speed (16,000 rpm, corresponding to 180 km/h) is marked in all figures in this paper.

## 2. Thermal Deformation of Deep Groove Ball Bearings in the High Speed Electric Drive System

**Figure 1:**
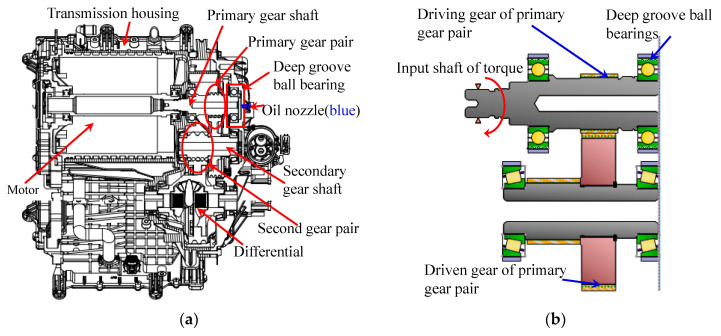
(**a**) Sectional view of the structure of an electric drive system and (**b**) the assembly drawing of primary gear pair.

The sectional view of a high speed electric drive helical gear drive system and a primary gear pair connected to the motor output shaft are shown in Figure 1, respectively. Firstly, the statics and pseudo-statics models of the deep groove ball bearing in the primary gear pair were established. Then, based on the thermal resistance network method, the deformation of the temperature rise of the ball bearing under the high speed working condition was obtained, which laid a foundation for the bearing stiffness calculation under the subsequent thermal deformation conditions.

### 2.1. Statics Analysis and Geometric Deformation Relationship of a Deep Groove Ball Bearing

**Figure 2:**
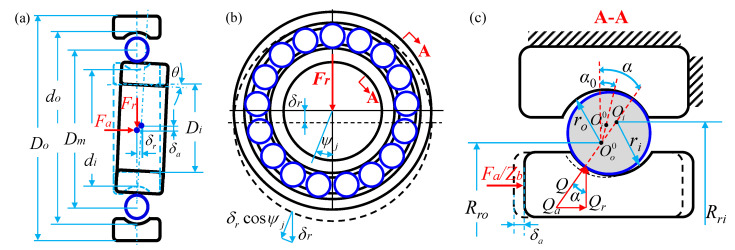
Geometric deformation of a deep groove ball bearing under different loads: (**a**) axial load, (**b**) radial load, and (**c**) torque load.

When the deep groove ball bearing was imposed under the different direction loads, the geometric deformation and force diagrams of bearing are shown in Figure 2, respectively. The point contact exists between the bearing ball with the inner ring and the outer ring, respectively. According to the Hertz contact theory [30,31], it is assumed that the elastic deformation is only generated by the contact, and the load is perpendicular to the contact surface with an oval shape, which is completely smooth without the tangential friction. Based on the least square method, the ellipticity *κ*, the first and the second types of complete elliptic integral Γ and Π can be obtained, respectively. The subscripts are *i* and *o*, and they represent the contact ellipse of the bearing ball with the inner and outer rings, respectively [32]. The contact stiffness between the ball and the inner ring (*K_i_*), and between the ball and the outer ring (*K_o_*) can be expressed as:

(1) $$\{\begin{array}{l} K_{i}=\frac{3}{2}{\left(\frac{\pi \kappa_{i}E_{i}^{\prime }}{3\Gamma_{i}}\right)}^{2/3}{\left(\frac{2\Pi_{i}}{\Gamma_{i}\sum \rho_{i}}\right)}^{1/3}Q_{i}^{1/3}\\ K_{o}=\frac{3}{2}{\left(\frac{\pi \kappa_{o}E_{o}^{\prime }}{3\Gamma_{o}}\right)}^{2/3}{\left(\frac{2\Pi_{o}}{\Gamma_{o}\sum \rho_{o}}\right)}^{1/3}Q_{o}^{1/3} \end{array}$$

It can be seen in Figure 2c that the contact load on the normal bearing ball is: *Q* = *F_a_*/(*Z_b_*sin*α*). The relation between the axial force of bearing and the deformation coefficient of contact load is denoted as [33]:

(2) $$\frac{F_{a}}{Z_{b}K_{n}{\left(B_{f}D_{b}\right)}^{1.5}}=\sin\alpha {\left(\frac{\cos\alpha_{0}}{\cos\alpha }-1\right)}^{1.5}$$

where, *F_a_* is the axial load (including the axial preload and axial load), *K_n_* is the bearing equivalent contact load deformation coefficient, proposed in Equation (3), *Z_b_* is the number of bearing balls, *B_f_* = *f_i_*+*f_o_*-1, is the total curvature, where *f_i_* = *r_i_*/*D_b_* and *f_o_* = *r_o_*/*D_b_* are the internal and external curvature coefficients, respectively, *r_i_* and *r_o_* are the radius values of curvature of inner and outer raceway, respectively, *D_b_* is the diameter of ball, and *α*_0_ is the initial contact angle of the bearing. The Newton–Raphson method was used to calculate the actual contact angle under the action of axial preload, and its iterative equation can be described in Equation (4).

(3) $$K_{n}={\left(K_{i}^{-2/3}+K_{o}^{-2/3}\right)}^{-3/2}$$

(4) $$\alpha^{\prime }=\alpha +\frac{\frac{F_{a}}{Z{D_{b}}^{2}K}-\sin\alpha {\left(\frac{\cos\alpha_{0}}{\cos\alpha }-1\right)}^{1.5}}{\cos\alpha {\left(\frac{\cos\alpha_{0}}{\cos\alpha }-1\right)}^{1.5}+1.5\tan^{2}\alpha {\left(\frac{\cos\alpha_{0}}{\cos\alpha }-1\right)}^{0.5}\cos\alpha_{0}}$$

**Figure 3:**
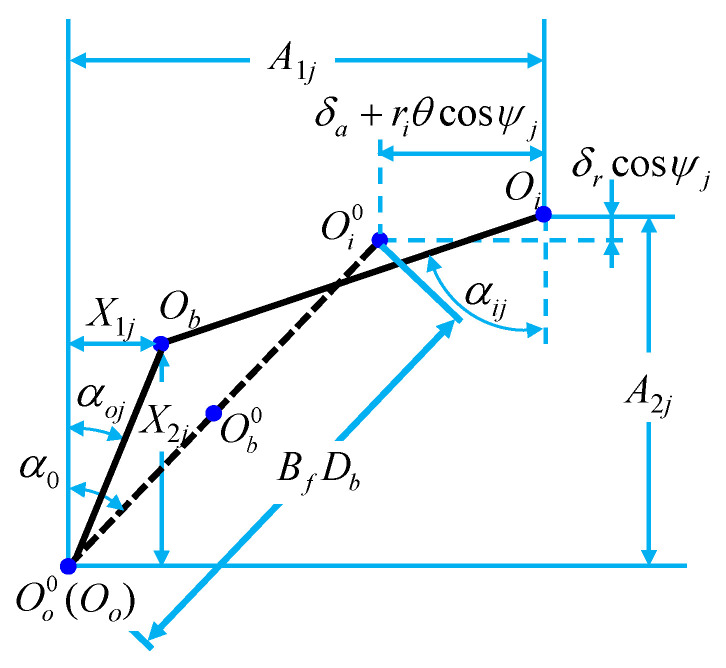
The contact angles, deformations, and displacements for the ball–raceway contacts at azimuth *ψ_j_* with the imposed load.

The deflection of the bearing around the *x* and *y* directions is ignored, but the deflection around the *z* direction is considered. In this case, the contact angles, deformations, and displacements for the ball–raceway contacts at azimuth *ψ_j_* are shown in Figure 3. Then, the axial and radial distances between the loci of inner and outer raceway groove curvature center (*A*_1*j*_ and *A*_2_) can be expressed as:

(5) $$\{\begin{array}{l} A_{1j}=B_{f}D_{b}\sin\alpha_{0}+\delta_{a}+R_{ri}\theta \cos\psi_{j}\\ A_{2j}=B_{f}D_{b}\cos\alpha_{0}+\delta_{r}\cos\psi_{j} \end{array}$$

(6) $$\{\begin{array}{l} R_{ri}=D_{m}/2+\left(f_{i}-0.5\right)D_{b}\cos\alpha \\ R_{ro}=D_{m}/2+\left(f_{o}-0.5\right)D_{b}\cos\alpha \end{array}$$

(7) $$\psi_{j}=2\pi \left(j-1\right)/Z_{b}$$

where, *δ_a_* and *δ_r_* are the relative displacements of the inner and outer ring under the action of external forces, respectively, and *θ* is the angle of the bearing under the external forces. *R_ri_* and *R_ro_* are the radius values to locus of inner and outer raceway groove curvature centers, respectively, and the calculation equation is shown in Equation (6). *ψ_j_* is the position angle of the *j*_th_ ball, and the calculation equation is shown in Equation (7).

### 2.2. Quasi-Statics Analysis of the High Speed Running Bearing and Its Deformation Geometry Equation

**Figure 4:**
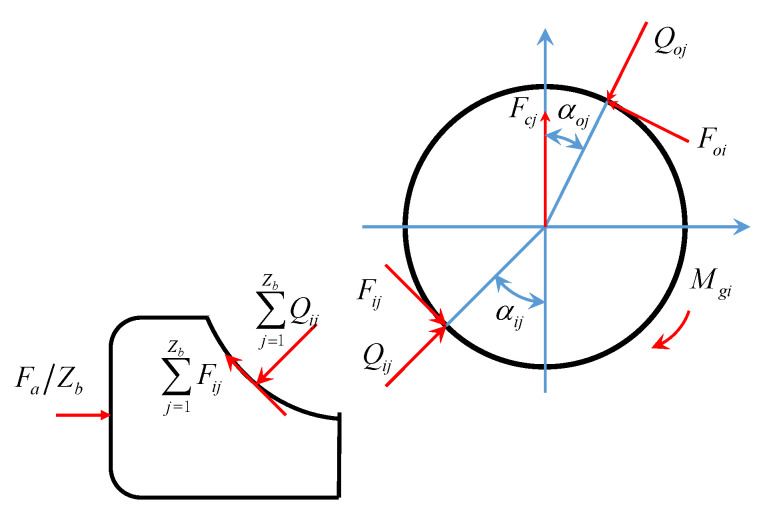
Ball loading at angular position *ψ_j_*.

Under the condition of high speed, the diameter of bearing inner groove and contact angle can be changed by the centrifugal force. Therefore, for the high speed electric drive system, it is necessary to analyze the deformation and stiffness of bearing under the high speed working condition. In order to simplify the calculation, the rotational slipping of the bearing ball is ignored and only the axial and radial forces of the bearing are considered in this study. Then, the force on the *j*_th_ ball at the high speed is shown in Figure 4 [34].

When the ball is subjected to the contact loads *Q_ij_* and *Q_oj_*, gyroscopic moment *M_gj_*, and the friction forces *F_ij_* and *F_oj_* generated between the inner and outer rings of the bearing, the equations of mechanical equilibrium under the load of the bearing and its ball can be obtained as:

(8) $$\{\begin{array}{l} F_{a}-\sum_{j=1}^{Z}\left(Q_{ij}\cos\alpha_{ij}+F_{ij}\cos\alpha_{ij}\right)=0\\ F_{r}-\sum_{j=1}^{Z}\left(Q_{ij}\cos\alpha_{ij}+F_{ij}\sin\alpha_{ij}\right)\cos\psi_{j}=0\\ M_{gi}-\sum_{j=1}^{Z}\left[\left(Q_{ij}\sin\alpha_{ij}+F_{ij}\cos\alpha_{ij}\right)R_{ri}-r_{i}F_{ij}\right]\cos\psi_{j}=0 \end{array}$$

where, *F_a_* is the axial force (including the preload *F_a_*_0_), *F_r_* is the radial force, *M_gi_* is the torque around the axial center of the bearing, and *α_ij_* and *α_ij_* represent the actual contact angle between the *j*_th_ bearing ball and the inner and outer rings of the bearing, respectively. According to the Hertz contact theory, the normal contact load between the ball and bearing inner or outer rings can be expressed as:

(9) $$\{\begin{array}{l} Q_{ij}=K_{ij}\delta_{ij}^{1.5}\\ Q_{oj}=K_{oj}\delta_{oj}^{1.5} \end{array}$$

where, *K_ij_* and *K_oj_* are the load deformation coefficients of the *j*_th_ bearing ball and the inner and outer ring, respectively. At the same time, according to the calculation equation [35], when the outer diameter of the thick-walled cylinder is under the pressure of *p*, the relative displacement of the inner and outer rings of the bearing under the centrifugal force can be calculated. The specific expression can be denoted as:

(10) $$u_{r}{|}_{r=D/2}=\frac{\left(1+\nu_{s}\right)D\left[D_{s}^{2}+\left(1-2\nu_{s}\right)D^{2}\right]}{2E_{s}\left(D_{s}^{2}-D^{2}\right)}p+\frac{\left(1+\nu_{s}\right)D\left[\left(2-3\nu_{s}\right)D_{s}^{2}-\left(1-2\nu_{s}\right)D^{2}\right]}{32E_{s}}\rho_{s}\omega^{2}$$

where *ρ_s_*, *v_s_*, *E_s_*, and *D_s_* are the material density, Poisson’s ratio, elastic modulus, and diameter of the transmission shaft, respectively, and *D* is the diameter of the connecting surface of the transmission shaft [35].

The axial and radial distances between the loci of inner and outer raceway groove curvature center (*A*_1*j*_ and *A*_2*j*_) can be expressed as:

(11) $$\{\begin{array}{l} A_{1j}=B_{f}D_{b}\sin\alpha_{0}+\delta_{a}+R_{ri}\theta \cos\psi_{j}\\ A_{2j}=B_{f}D_{b}\cos\alpha_{0}+\delta_{r}\cos\psi_{j}+u_{r} \end{array}$$

where, compared with the static load, the vertical distance increases the displacement *u_r_* caused by the centrifugal force. According to the geometrical relationship, the contact angles between the ball and the inner and outer raceway are expressed as:

(12) $$\{\begin{array}{l} \alpha_{oj}=\arccos\left(\frac{X_{2j}}{(f_{o}-0.5)D_{b}+\delta_{oj}}\right)\\ \alpha_{ij}=\arcsin\left(\frac{A_{1j}-X_{1j}}{(f_{i}-0.5)D_{b}+\delta_{ij}}\right) \end{array}$$

where, *X*_1*j*_ and *X*_2*j*_, are the axial and radial projection values of distance between ball center and outer raceway center, respectively, and *δ_ij_* and *δ_oj_* are the elastic deformation of the inner and outer ring in contact with the ball, respectively.

**Figure 5:**
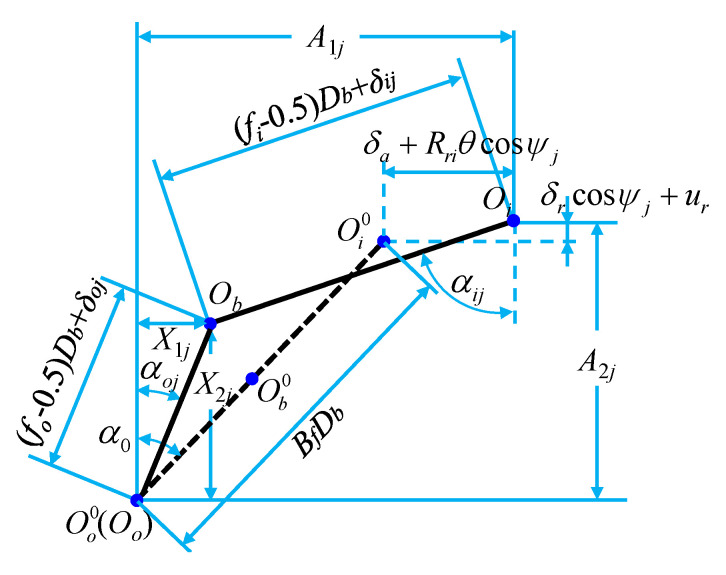
The contact angles, deformations, and displacements for the ball–raceway contacts at azimuth *ψ_j_* with the working load and centrifugal force.

Combining Figure 5 and Pythagorean Theorem, the compatible equation of deformation geometry of contact between the ball and the inner and outer raceways can be obtained by:

(13) $$\{\begin{array}{l} {\left(A_{1j}-X_{1j}\right)}^{2}+{\left(A_{2j}-X_{2j}\right)}^{2}={\left[(f_{i}-0.5)D_{b}+\delta_{ij}\right]}^{2}\\ X_{1j}^{2}+X_{2j}^{2}={\left[(f_{i}-0.5)D_{b}+\delta_{oj}\right]}^{2} \end{array}$$

According to Figure 4 and Figure 5, considering the centrifugal and gyro effects during the high speed operation of the bearing [33], the equilibrium equations of the bearing ball are obtained as follows:

(14) $$\{\begin{array}{l} Q_{ij}\sin\alpha_{ij}-Q_{oj}\sin\alpha_{oj}+F_{ij}\cos\alpha_{ij}-F_{oj}\cos\alpha_{oj}=0\\ Q_{ij}\cos\alpha_{ij}-Q_{oj}\cos\alpha_{oj}-F_{ij}\sin\alpha_{ij}+F_{oj}\sin\alpha_{oj}+F_{cj}=0 \end{array}$$

where, the friction forces *F_ij_* and *F_oj_* are related to the gyroscopic moment *M_gj_* of the bearing ball. Meanwhile, the torque caused by the friction is balanced with the torque caused by the gyroscopic moment, and Equation (14) can be expressed as:

(15) $$\{\begin{array}{l} Q_{ij}\sin\alpha_{ij}-Q_{oj}\sin\alpha_{oj}+\lambda_{ij}\frac{M_{gj}}{D_{b}}\cos\alpha_{ij}-\lambda_{oj}\frac{M_{gj}}{D_{b}}\cos\alpha_{oj}=0\\ Q_{ij}\cos\alpha_{ij}-Q_{oj}\cos\alpha_{oj}-\lambda_{ij}\frac{M_{gj}}{D_{b}}\sin\alpha_{ij}+\lambda_{oj}\frac{M_{gj}}{D_{b}}\sin\alpha_{oj}+F_{cj}=0 \end{array}$$

The centrifugal force, generated by the high speed operation of the bearing, makes the ball purely roll on the bearing outer raceway. Thus, in Equation (15), *λ_ij_* = 0 and *λ_oj_* = 2 [31]. *F_cj_* is the ball centrifugal force, and it can be expressed as:

(16) $$F_{cj}=\frac{\pi \rho_{b}D_{b}^{2}D_{m}\omega_{cj}^{2}}{12}$$

where, *ρ_b_* is the density of ball, and *ω_cj_* is the ball orbit velocity. The calculation equation of gyroscopic moment is denoted as follows:

(17) $$M_{gj}=J_{b}\omega_{cj}\omega_{bj}\sin\beta_{j}$$

where *ω_bj_* is the spinning speed of ball, *J_b_* is the mass moment of inertia of ball, proposed in Equation (18), and *β_j_* is the attitude angle of the *j*_th_ ball, as shown in Equation (19) [33].

(18) $$J_{b}=\frac{\rho_{b}\pi D_{b}^{5}}{60}$$

(19) $$\beta_{j}=\arctan\frac{\sin\alpha_{oj}}{\cos\alpha_{oj}+\gamma^{\prime }}$$

where, *γ’* = *D_b_*/*D_m_* is the ratio of the ball diameter to the pitch diameter of bearing.

### 2.3. Calorific Value Calculation Model of the Deep Groove Ball Bearing Running at the High Speed

Under the high speed working conditions, in addition to the thermal deformation as a result of the centrifugal force, the bearing will also produce a lot of heat and thermal deformation resulting from the friction. According to Palmgren [36] test analysis, the friction torque is mainly generated by the viscosity of lubricant and the effect of loading. At the same time, the ball will produce the spinning motion in the outer raceway [33], which will cause the friction torque and generate the heat. Therefore, the heat produced by the friction torque, which is caused by the bearing load, lubrication viscosity, and spinning motion, is mainly considered in the heat calculation.

The friction torque *M*_1_ generated by the bearing load can be obtained by:

(20) $$M_{1}=f_{1}P_{1}D_{m}$$

where, *f*_1_ is the coefficient related to the bearing type and loads [36]. *P*_1_ is the force related to the magnitude and the directions of axial and radial loads [36].

The viscous friction moment *M_μ_* caused by the orbiting rolling elements as they plow through the viscous lubricant can be expressed as [36]:

(21) Mμ={10-7f0(μn)23Dm3μn≥20001.6×10-5f0Dm3μn<2000

where *f*_o_ is a factor depending on the type of the bearing and the method of lubrication. Since the lubrication method is oil bath or oil mist, so *f_o_ =* 1−2 [37]. *n* is the bearing speed and *μ* is the lubricant kinematic viscosity at the working temperature.

The magnitude of the spinning moment of the *j*_th_ ball can be obtained by [38]:

(22) $$\{\begin{array}{l} M_{sij}=\frac{3}{8}\mu_{si}Q_{ij}A_{ij}\Pi_{i}\\ M_{soj}=\frac{3}{8}\mu_{so}Q_{oj}A_{oj}\Pi_{o} \end{array}$$

where, *μ_si_* and *μ_so_* are the coefficients of friction between the ball and inner and outer raceway, respectively. *Q_ij_* and *Q_oj_* are the ball loads between the *j*_th_ ball and inner and outer raceway, respectively. *A_ij_* and *A_oj_* are the semimajor axis of projected contact ellipse between the *j*_th_ ball and the inner and outer raceway, respectively. Π*i* and Π*o* are the complete elliptic integral of the second kind with the modulus.

Hence, the total heat generated by the total friction torque can be denoted as:

(23) $$H=\frac{\pi n}{30}\left(M_{1}+M_{\mu }\right)+\omega_{si}M_{si}+\omega_{so}M_{so}$$

where, *ω_si_* and *ω_so_* are the spinning of the outer raceway relative to the ball, and they can be calculated by:

(24) $$\{\begin{array}{l} \omega_{so}=\omega_{b}\left(\sin\alpha_{o}\cos\beta -\cos\alpha_{o}\sin\beta \right)-\omega_{o}\sin\alpha_{o}\\ \omega_{si}=-\omega_{b}\left(\cos\alpha_{i}\sin\beta -\sin\alpha_{i}\cos\beta \right)+\omega_{i}\sin\alpha_{i} \end{array}$$

where, *ω_i_* and *ω_o_* are the relative angular speeds of the inner and outer raceway, respectively. *ω_b_* is the spinning speed of ball, which is calculated by Equation (25), *ω_c_* is the rotational speed referring to the orbital motion, and it can be calculated by Equation (26), *ω_n_* is the absolute angular speed of the inner raceway, and *β* is the attitude angle of the ball, as shown in Equation (19).

(25) $$\omega_{b}=\omega_{c}\frac{\left(D_{m}/D_{b}+\cos\alpha_{o}\right)}{\left(\cos\beta \cos\alpha_{i}+\sin\beta \sin\alpha_{o}\right)}$$

(26) $$\omega_{c}=\omega_{n}{\left(1+\frac{\left(\frac{D_{m}}{D_{b}}+\cos\alpha_{o}\right)\left(\cos\beta \cos\alpha_{i}+\sin\beta \sin\alpha_{i}\right)}{\left(\frac{D_{m}}{D_{b}}+\cos\alpha_{i}\right)\left(\cos\beta \cos\alpha_{o}+\sin\beta \sin\alpha_{o}\right)}\right)}^{-1}$$

**Figure 6:**
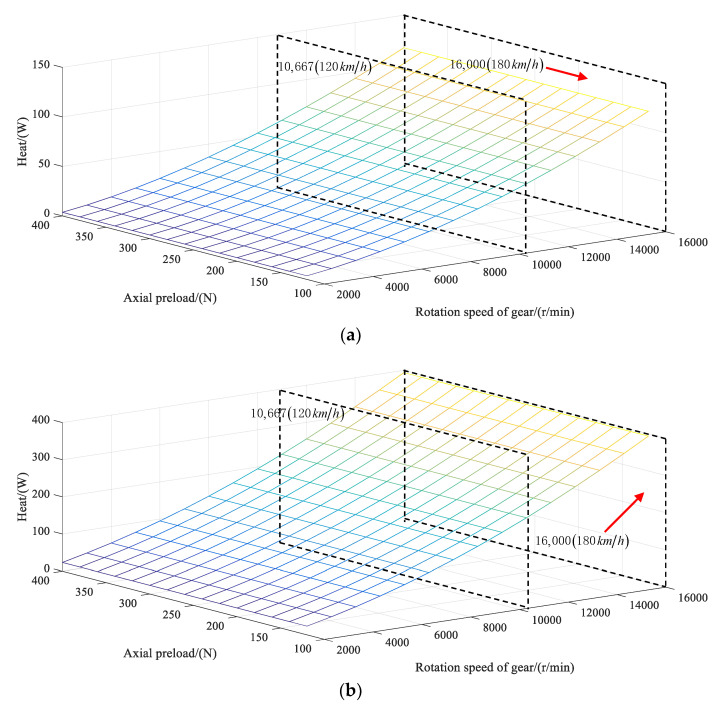
Calorific values of bearing under different loading conditions: (**a**) no load and (**b**) the load with the input torque of 100 Nm.

Combining the above equations and the specific parameters of bearing in Table 1, the calorific values can be obtained under different rotating speeds and preload values with no-load and load conditions, as shown in Figure 6a,b, respectively, ignoring the influence of time-varying meshing force of gear on the bearing characteristics. The imposed load is 100 Nm, and the axial and radial forces are 2380.9 N and 1189.9 N, respectively, at this moment.

In Figure 6, it is found that the heat generation significantly improves with the increase of rotating speed under the same axial preload. At the same speed, the heat generation also increases with the increase of axial preload.

The bearing frictional heat generation will radiate outwards in the form of heat conduction and convection. According to the research conclusions of Burton and Staph [39], after the heat generation of bearing, half of the heat enters the ball and the other half enters the inner and outer rings. Therefore, the obtained heat of the inner ring (*H_i_*), the obtained heat of the outer ring (*H_o_*), and the obtained heat of the ball (*H_b_*) are calculated as follows:

(27) $$\{\begin{array}{l} H_{i}=0.25\left(\pi n\left(M_{1}+M_{\nu }\right)/30\right)+0.5\left(\omega_{si}M_{si}\right)\\ H_{o}=0.25\left(\pi n\left(M_{1}+M_{\nu }\right)/30\right)\\ H_{b}=0.5\left(\pi n\left(M_{1}+M_{\nu }\right)/30\right)+0.5\left(\omega_{si}M_{si}\right) \end{array}$$

### 2.4. Temperature Rise and Thermal Deformation of the Bearing Based on the Thermal Resistance Network Method

**Figure 7:**
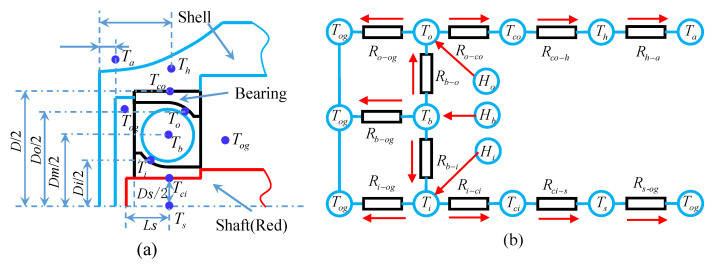
(**a**) Thermal node distribution and (**b**) thermal network model of the deep groove ball bearing.

After the generation of heat in the bearing, the heat will be transferred outwards, which will improve the temperature of the inner and outer rings, and the bearing balls. The axial and radial thermal deformation will occur between the inner and outer rings of the bearing and change the contact angle of the inner and outer rings of the bearing. Under the stable working condition, the temperature difference between bearing, gearbox, and shaft system is small, and the heat radiation effect on the thermal behaviors of bearings is little, so that the influence of heat radiation can be ignored [40]. Thermal nodes are set at different points of the bearing and connected as a heat network in the form of heat resistance, as shown in Figure 7.

In Figure 7b, *R_ci-s_* is the heat conduction resistance of the shaft, which is transferred from the contact point between the bearing inner ring and the transmission shaft to the thermal node of transmission shaft. *R_i-ci_* is the heat conduction resistance of the inner ring of the bearing, which is transferred from the contact point between the bearing inner ring and ball to the contact point between the bearing inner ring and the transmission shaft. *R_b-i_* is the heat conduction resistance of the ball, which is transferred from the ball to the contact point between the inner ring and the ball. *R_b-o_* is the heat conduction resistance of the ball, which is transferred from the ball to the contact point between the outer ring and the ball. *R_o-co_* is the heat conduction resistance of the outer ring, which is transferred from the contact point between the ball and the outer ring to the contact point between the bearing seat and the outer ring. *R_co-h_* is the heat conduction resistance of the bearing seat, which is transferred from the contact point between the outer ring and the bearing seat to the thermal node of the bearing seat. *R_h_*_-*a*_ is the convective heat resistance between the bearing seat and the air, which is transferred from the bearing seat to the air. *R_s-og_* is the convective heat resistance which is used to depict the heat transfer between transmission shaft and the oil–gas mixture. The heats are transmitted from the inner ring to oil–gas mixture by the resistances *R_i-og_.* The heat is transmitted from balls to oil–gas mixture by the corresponding resistance *R_b-og_. R_o-og_* is the convective heat resistance which is used to depict the heat transfer between balls and oil–gas [40,41]. The lubrication method of the system is to force the lubrication through oil injection at the end cover of the bearing as shown the oil nozzle (blue) in Figure 1. In the steady-state heat transfer process, according to Kirchhof’s energy balance principle, the equations of heat balance at each node were established. Therefore, the heat balance equations of the bearing model were established to analyze and calculate the temperature between each part of the bearing. To simplify the process, the following assumptions were made:

(1) The heat transfer process of the bearing is steady;
(2) The three-dimensional heat transfer model is simplified into a one-dimensional model radial heat transfer [35,40,42], and the heat generation in the contact zone is caused by friction losses and rolling resistance;
(3) The influence of temperature rise on the lubricating oil performance is ignored;
(4) The flow rate of the cooling fluid is large enough so that the fluctuating temperature of the fluid is not considered, and the temperature of the oil–gas mixture is assumed to be fixed at 28 °C;
(5) The material of all parts is assumed to be isotropic.

Then, the thermal balance equation of the system can be established as follows:

(28) $$\{\begin{array}{l} \frac{T_{s}-T_{og}}{R_{s-og}}-\frac{T_{ci}-T_{s}}{R_{ci-s}}=0\\ \frac{T_{ci}-T_{s}}{R_{ci-s}}-\frac{T_{i}-T_{ci}}{R_{i-ci}}=0\\ \frac{T_{i}-T_{ci}}{R_{i-ci}}+\frac{T_{i}-T_{b}}{R_{b-i}}+\frac{T_{i}-T_{og}}{R_{i-og}}=0.5H_{i}\\ \frac{T_{b}-T_{og}}{R_{b-og}}+\frac{T_{i}-T_{b}}{R_{b-i}}+\frac{T_{o}-T_{b}}{R_{b-o}}=0.5H_{i}+0.5H_{o}\\ \frac{T_{o}-T_{b}}{R_{b-o}}+\frac{T_{o}-T_{co}}{R_{o-co}}+\frac{T_{o}-T_{og}}{R_{o-og}}=0.5H_{o}\\ \frac{T_{co}-T_{h}}{R_{co-h}}+\frac{T_{o}-T_{co}}{R_{o-co}}=0\\ \frac{T_{h}-T_{a}}{R_{h-a}}+\frac{T_{co}-T_{h}}{R_{co-h}}=0 \end{array}$$

where, *T_s_* is the temperature of the transmission shaft node, *T_ci_* is the temperature of the node at the contact point between the inner ring of the bearing and the shaft, *T_og_* is the temperature of the gas–oil mixture, *T_i_* is the temperature of the inner raceway node, *T_b_* is the temperature of the ball node, *T_o_* is the temperature of the outer raceway node, *T_co_* is the temperature at the contact point between the outer ring and the bearing seat, and *T_a_* is the air temperature. The force oil cooling system, which is applied in Figure 1, can help to contain the temperature of the system and reduce the error as described in [40,43]. In addition, the fluctuating temperature of the fluid was neglected, as mentioned in assumption (4). Therefore, the temperature of the oil–gas mixture *T_og_* which set fixed at 28 °C. The temperature difference between the two nodes is expressed as:

(29) $$\Delta T=\frac{k_{k}\cdot S}{L\cdot H}$$

where, *T* is the temperature difference between two nodes, *H* is the transfer of heat, *k_k_* is the thermal conductivity of the material, *S* is the area perpendicular to the direction of heat flow between two points, and *L* is the distance between two nodes. Therefore, the thermal conductivity equation between bearing rings can be expressed as:

(30) $$H_{q}=\frac{2\pi kC}{\ln\left(D_{o}/D_{i}\right)}\left(T_{o}-T_{i}\right)$$

The heat conduction is related to the thermal conductivity of the material, while the heat convection is related to the heat transfer coefficient of the medium surface. Then, the heat transfer coefficient of the air and the surface of lubricating oil can be denoted as, respectively:

(31) $$\{\begin{array}{l} h_{a}=2.3\times 10^{-5}{\left(T_{1}-T_{2}\right)}^{0.25}\\ h_{og}=0.332k_{v}P_{\gamma }^{1/3}{\left(\frac{n_{v}}{2r_{x}}\right)}^{0.5} \end{array}$$

where, *k_v_* is the thermal conductivity of lubricating oil, *P_γ_* is Prandtl number of lubricating oil, *n_v_* is one third of the speed of the cage, *μ* is kinematic viscosity, and *r_x_* is the radius of the raceway.

According to the definition of thermal resistance, the above equation can be converted into:

(32) $$R=\frac{\left(T_{o}-T_{i}\right)}{q}=\frac{\ln\left(D_{o}/D_{i}\right)}{2\pi kC}$$

where, *q* is the heat; *C* is the width of the outer ring. *D_o_* and *D_i_* are the diameters of the inner and outer rings, respectively.

The thermal resistance of each structure in the process of heat transfer is shown in Table 2. k_i_, k_o_, k_b_, k_s_, and k_h_ are the thermal conductivity of the inner ring, the outer ring, the ball, the shaft, and the bearing seat, respectively. h_i_, h_o_, h_s_, h_b_, h_h_ are the coefficients of free/force convection heat transfer [36,40]. D_s_ and D_h_ are the outer diameters of the shaft and the distance of the bearing seat from the center line of the shaft, respectively. B, C, L_s_, and L_h_ are the width of bearing inner ring and outer ring, the distance between the node T_s_ from the transmission shaft end, the height of bearing housing, respectively, as shown in Figure 7.

Combining the proposed heat balance equation of the bearing and the heat resistance of each node, the Newton–Raphson iterative method was used to solve the heat balance equation, assuming that the initial ambient temperature is 26 °C, and the temperature of each node was obtained, satisfying the setting accuracy.

**Figure 8:**
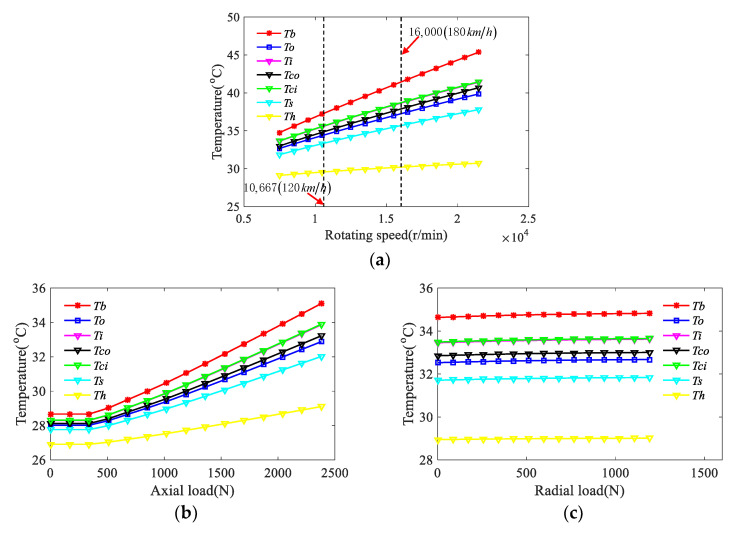
Temperature curve diagram of each node of the thermal network changing with the rotating speed and load: (**a**) node temperature variation diagram with the rotating speed under no-load, (**b**) node temperature variation diagram with axial load, and (**c**) node temperature variation diagram with radial load.

Under the condition of axial preload force of 300 N, the temperature of each node was calculated under the conditions of no-load (with the range of rotating speed from 7250 to 22,500 r/min), and the different radial and axial loads. For example, when the rotating speed is 6500 r/min, the constant radial load is 1189.9 N, and the range of axial load is from 0 to 2380.9 N. On the contrary, the axial load is constant while the radial loads are various (with the rotating speed of 6500 r/min, the axial load of 2380.9 N, and the range of radial load from 0 to 1189.9 N), and the calculation results are shown in Figure 8.

According to Figure 8a, when there is no load, the temperature of each node increases almost linearly with the increase of rotating speed. Figure 8b,c indicate that, with the increase of the axial or radial load, the temperature of each node increases to a certain extent, but the influence of axial load on the node temperature is much greater than that of radial load. In the same working condition, the temperature rise of the bearing ball is the maximum and that of the housing is the minimum in each node of the bearing. The temperature at the contact point of the inner and outer raceway is similar with that of the inner and outer rings of the bearing, and the temperatures at the contact point and the thermal node of inner ring are higher than that of the outer ring.

According to the temperature rise of the bearing, the corresponding thermal deformation can be calculated by [40,41,42,44]:

(33) $$u_{T}=a_{x}\Delta Td$$

where, *u_T_* is the thermal deformation, and *a_x_* is the thermal expansion coefficient of the material, where subscript *x* = *s*, *i, o,* and *h*, respectively, refer to the thermal expansion coefficients of the transmission shaft, inner ring, outer ring, and transmission housing. △*T* = *T*_1_ − *T*_2_ is the temperature gap of the part and *d* is the diameter of each part. Due to the axisymmetric distribution of bearing temperature, depending on the stress–strain relationship [45], *u_Ts_* which stands for the thermal deformation of transmission shaft, *u_Ti_* which is the thermal deformation of bearing inner raceway, *u_Ti_^’^* which is the radial thermal deformation of inner ring, *u_To_* which represents the deformation of the outer raceway, *u_To_^’^* which denotes the radial thermal deformation of outer ring, *u_Tb_* which is the thermal deformation of the ball, and *u_Th_* which stands for the radial thermal deformation of the bearing seat, can be obtained by, respectively:

(34) $$\{\begin{array}{l} u_{Ts}=a_{s}\Delta T_{s}\left(1+v_{s}\right)D_{s}\\ u_{Ti}=a_{i}\Delta T_{i}d_{i}\\ {u_{Ti}}^{\prime }=a_{i}\Delta T_{i}D_{i}\\ u_{To}=a_{o}\Delta T_{o}d_{o}\\ {u_{To}}^{\prime }=a_{o}\Delta T_{o}D_{o}\\ u_{Tb}=a_{o}\Delta T_{b}D_{b}\\ u_{Th}=a_{h}\Delta T_{h}\left(1+v_{h}\right)D_{h} \end{array}$$

Based on Equation (34) and bearing temperature rise model, the radial thermal deformation equations of inner raceway and outer raceway can be obtained [32], where the thermal deformation of the inner raceway is considering the effect of the thermal deformation of the shaft, and the thermal deformation of the outer raceway is considering the deformation effect of the bearing seat:

(35) $$\{\begin{array}{l} u_{Tri}=u_{Ti}\frac{\left(u_{Ts}-{u_{Ti}}^{\prime }\right)D_{Ti}}{D_{Ts}}=a_{i}\Delta T_{i}D_{i}+\left(a_{s}\Delta T_{s}\left(1+\nu_{s}\right)D_{s}-a_{i}\Delta T_{i}D_{i}\right)\frac{D_{i}}{D_{s}}=a_{s}\Delta T_{s}\left(1+\nu_{s}\right)D_{i}\\ u_{Tro}=u_{T_{i}}+\frac{\left(u_{Th}-{u_{To}}^{\prime }\right)D_{o}}{D}=a_{o}\Delta T_{o}D_{o}+\frac{\left(a_{h}\Delta T_{h}\left(1+\nu_{h}\right)D-a_{o}\Delta T_{o}D\right)D_{o}}{D}=a_{h}\Delta T_{h}\left(1+\nu_{h}\right)D_{o} \end{array}$$

Considering the ball deformation, *u_Tr_* which refers to the relative radial thermal deformation between the inner and outer groove raceway can be expressed as:

(36) $$u_{Tr}=u_{Tri}-u_{Tro}-2u_{Tb}=a_{s}\Delta T_{s}\left(1+v_{s}\right)D_{s}+a_{h}\Delta T_{h}\left(1+v_{h}\right)D_{o}-2a_{b}\Delta T_{b}D_{m}$$

Considering the deformation of the transmission shaft and the bearing seat, *u_Ta_* which refers to the axial thermal deformation between the inner and outer ring raceway can be expressed as:

(37) $$u_{Ta}=\left(a_{h}\Delta T_{h}L_{h}-a_{s}\Delta T_{s}L_{s}\right)/2$$

**Figure 9:**
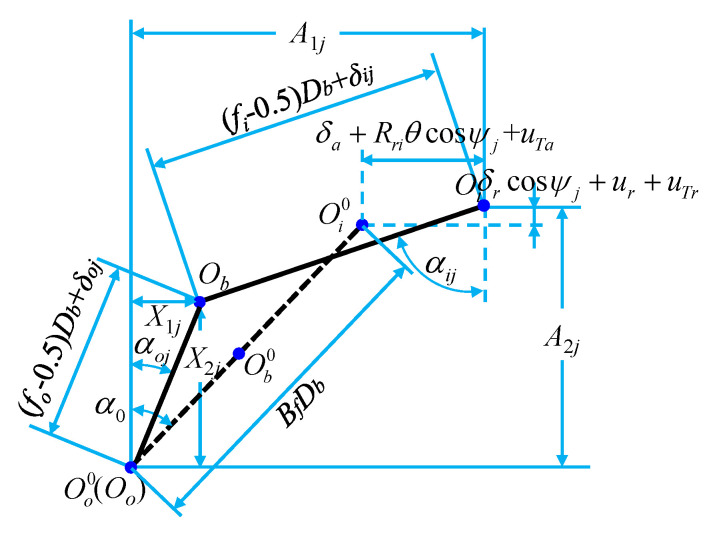
The contact angles, deformations, and displacements for the ball–raceway contacts at azimuth *ψ_j_* with the working load, centrifugal force, and thermal deformation.

When the thermal deformation is caused by the heat generation of the bearing, the axial and radial distance between the loci of inner and outer raceway groove curvature center changes, as shown in Figure 9. In this case, *A*_1*j*_ and *A*_2*j*_ can be expressed as:

(38) $$\{\begin{array}{l} A_{1j}=BD_{b}\sin\alpha_{0}+\delta_{a}+R_{ri}\theta \cos\psi_{j}+u_{Ta}\\ A_{2j}=BD_{b}\cos\alpha_{0}+\delta_{r}\cos\psi_{j}+u_{r}+u_{Tr} \end{array}$$

In addition to the relative displacement and angle under loading, the thermal deformations of the bearing, shaft, and bearing seat can also change the axial projection of distance between the ball center and the outer raceway groove curvature center.

**Figure 10:**
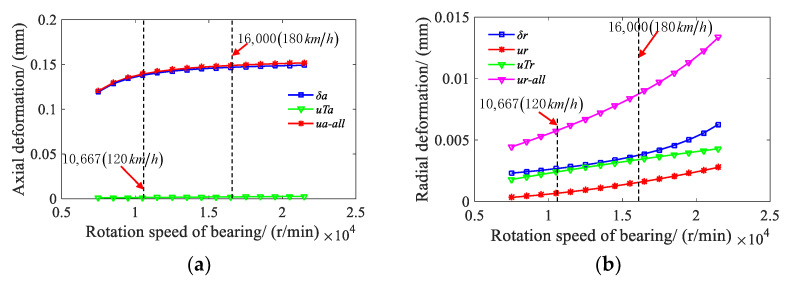
(**a**) Axial deformation displacement graph and (**b**) radial deformation displacement graph of the deep groove ball bearing under the centrifugal effect and thermal deformation.

With the preload of 300 N, the axial load of 2380.9 N, and the radial load of 1189.9 N, the relationship between the rotating speed and the axial deformation or the radial deformation of the bearing is shown in Figure 10. In Figure 10a, *u_a_*-*_all_* and *u_r_*-*_all_* represent the combined axial and radial deformation of the bearing, respectively. It can be seen from Figure 10 that the thermal and comprehensive deformations in axial and radial directions both improve with the increase of rotating speed, where the deformation is under loading. The axial thermal deformation of the bearing is far less than the axial deformation under loading, and it can be ignored. The radial thermal deformation of the bearing is close to that under loading, and it is greater than that of the centrifugal effect. The influence of the radial thermal deformation on the bearing dynamic characteristics should be considered.

## 3. Analysis on the Influence of Bearing Stiffness on the Nonlinear Dynamic Characteristics of the Gear Transmission System

### 3.1. Calculation of Bearing Stiffness under the Thermal Deformation Condition

The bearing stiffness affects the vibration characteristics and nonlinear behavior of the gear transmission system [29]. The better bearing stiffness may make the drive system run more smoothly. Based on the thermal deformation of the deep-groove ball bearing under the conditions of high speed operation, the bearing stiffness under the thermal deformation was calculated, and the nonlinear dynamic characteristics of the high speed electric drive gear transmission system were analyzed under the thermal deformation.

Firstly, based on Equations (13) and (15), the Newton–Raphson method was used to calculate the inner and outer ring loads (*Q_ij_* and *Q_oj_*), and the contact angles (*α_ij_* and *α_oj_*), meeting the accuracy requirements before and after the thermal deformation of the bearing. Then, the contact stiffness of the bearing under the condition of thermal deformation was calculated, and the axial and radial stiffness values were solved. The contact stiffness between the ball and inner or outer ring of the bearing is shown in Equation (1). The total axial stiffness of the bearing is the parallel of the axial contact stiffness between the balls and inner and outer rings, which can be expressed as:

(39) $$K_{a}=\sum_{j=1}^{Z_{b}}\frac{K_{aij}\times K_{aoj}}{K_{aij}+K_{aoj}}$$

where, *K_aij_* and *K_aoj_* are the axial contact stiffness values between the *j*_th_ ball and inner and outer rings, respectively, and they can be obtained by:

(40) $$\{\begin{array}{l} K_{aij}=K_{ij}\sin^{2}\alpha_{ij}\\ K_{aoj}=K_{oj}\sin^{2}\alpha_{oj} \end{array}$$

Similarly, the total radial stiffness of a bearing can be expressed as:

(41) $$K_{r}=\sum_{j=1}^{Z_{b}}\frac{K_{rij}\times K_{roj}}{K_{rij}+K_{roj}}\cos^{2}\left(\frac{2\pi \left(j-1\right)}{Z}\right)$$

where, *K_rij_* and *K_roj_* are the radial contact stiffness values between the *j*_th_ ball and inner and outer rings, respectively, denoted as:

(42) $$\{\begin{array}{l} K_{rij}=K_{ij}\cos^{2}\alpha_{ij}\\ K_{roj}=K_{oj}\cos^{2}\alpha_{oj} \end{array}$$

**Figure 11:**
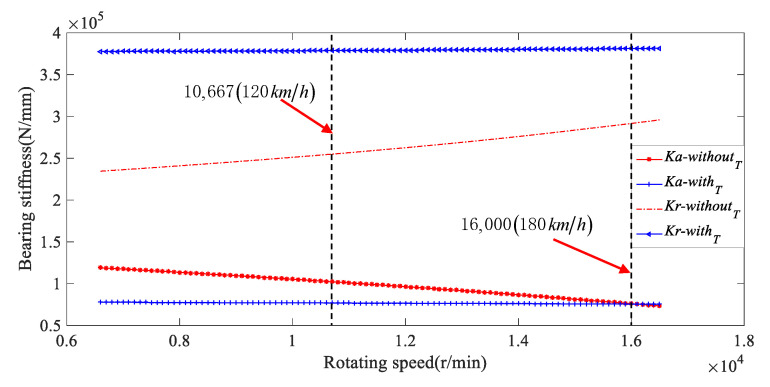
Axial and radial stiffness of the bearing with or without the thermal deformation.

Figure 11 shows the influence of the thermal deformation on the axial and radial stiffness of the bearing under the accelerated input torque of 100 Nm, where the range of acceleration speed is from 6500 to 16,500 r/min. The red ‘*’ shaped line and red dotted line represent the relationship curves of axial stiffness and radial stiffness versus the rotating speed, respectively, without considering the bearing temperature rise. The blue ‘+’ and blue ‘△’ shaped lines are the curves of axial stiffness and radial stiffness versus the rotating speed, respectively, considering the bearing temperature during the working conditions. In Figure 11, regardless of the thermal deformation of the bearing, the radial stiffness of the bearing slowly improves with the increase of the rotating speed, while the axial stiffness slightly decreases. This reason is that the centrifugal force and contact load of the bearing ball are improved, and the contact angle between the ball and the outer ring groove is reduced with the increase of the rotating speed. In addition, the thermal deformation of the bearing will reduce the radial clearance of the bearing and the contact angle of the outer ring at the same rotating speed, thus further improving the radial stiffness and reducing the axial stiffness of the bearing. However, the thermal deformation of the bearing results in the increase of the ball radius with the increase of rotating speed, and the contact stiffness and contact angle change under the deformation of the inner ring. The increase degree of the radial stiffness and the decrease degree of the axial stiffness may reduce with the increase of the rotating speed. Therefore, the effect of high speed and the influence of bearing temperature rise on the bearing stiffness should be considered when analyzing the nonlinear dynamic characteristics of the high speed electric drive gear system.

### 3.2. Dynamic Modeling of the High Speed Electric Drive Gear Transmission System

**Figure 12:**
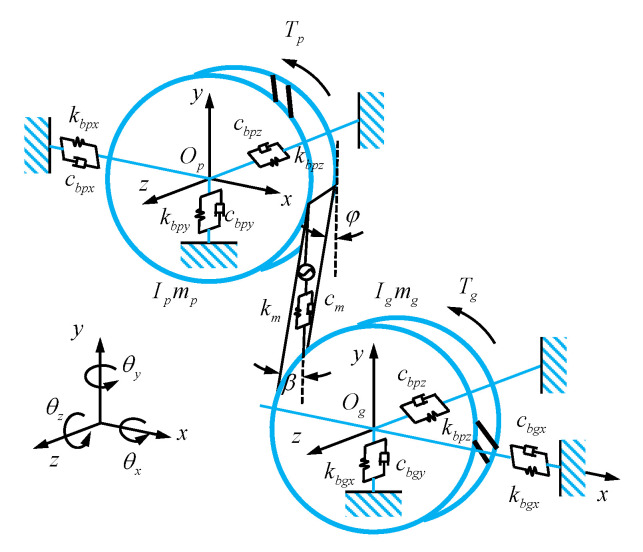
Dynamic model of the electric drive transmission system.

Based on the above analysis and calculation of bearing stiffness, a dynamic model of the helical gear system supported by the bearing was established, as shown in Figure 12.

In Figure 12, *O_p_* and *O_g_* are the rotation centers of the driving and driven gears, respectively, with the counterclockwise rotation around the central axis as the positive direction, and the compression spring stiffness direction as the positive direction of the meshing force. The stiffness/mass matrix of the shaft parts is extracted by the lumped-mass method, and the dynamic equations including the movement direction of the driving and driven gears along *x*, *y*, and *z* directions and the rotation direction around the axis of the gear can be established, respectively, as shown in Equations (43) and (44), to study the nonlinear dynamic characteristics of the gear system under different input torques and rotating speeds.

(43) $$\{\begin{array}{l} m_{p}{\ddot{x}}_{p}+c_{bpx}{\dot{x}}_{p}+k_{bpx}x_{p}+F_{n}\cos\beta_{g}\sin\varphi =0\\ m_{p}{\ddot{y}}_{p}+c_{bpy}{\dot{y}}_{p}+k_{bpy}y_{p}+F_{n}\cos\beta_{g}\cos\varphi =0\\ m_{p}{\ddot{z}}_{p}+c_{bpz}{\dot{z}}_{p}+k_{bpz}z_{p}+F_{n}\sin\beta_{g}=0\\ I_{p}{\ddot{\theta }}_{p}-t_{p}+F_{n}\cos\beta_{g}R_{bp}=0 \end{array}$$

(44) $$\{\begin{array}{l} m_{g}{\ddot{x}}_{g}+c_{bgx}{\dot{x}}_{g}+k_{bgx}x_{g}-F_{n}\cos\beta_{g}\sin\varphi =0\\ m_{g}{\ddot{y}}_{g}+c_{bgy}{\dot{y}}_{g}+k_{bgy}y_{g}-F_{n}\cos\beta_{g}\cos\varphi =0\\ m_{g}{\ddot{z}}_{g}+c_{bgz}{\dot{z}}_{g}+k_{bgz}z_{g}-F_{n}\sin\beta_{g}=0\\ I_{g}{\ddot{\theta }}_{g}+t_{g}-F_{n}\cos\beta_{g}R_{bg}=0 \end{array}$$

where, *m_p_* and *m_g_* are the mass values of driving and driven gears, respectively. *k_bpx_*, *k_bpy_*, *k_bpz_*, *k_bgx_*, *k_bgy_*, and *k_bgz_* are the supporting stiffness values of the bearing mounted on the driving and driven gear shafts in the *x*, *y*, and *z* directions, respectively. *c_bpx_*, *c_bpy_*, *c_bpz_*, *c_bgx_*, *c_bgy_*, and *c_bgz_* are the damping coefficients of the bearing mounted on the driving and driven gear shafts in the *x*, *y*, and *z* directions, respectively. *F_n_* is the meshing force. *I_p_* and *I_g_* are the moment values of inertia of the driving and driven gears, respectively; *R_bp_* and *R_bg_* are the base circle radius values of the driving and driven gears, respectively. *θ_p_* and *θ_g_* are the rotation angles of the driving and driven gears, respectively; *t_p_* and *t_g_* are input and output torques, respectively. *β_g_* is the helix angle and *φ* is the angle between the action surface and the vertical direction.

The meshing forces in the *x*, *y*, and *z* directions and the relative displacement in the normal direction can be expressed as:

(45) $$\{\begin{array}{l} \begin{array}{l} \delta_{x}=x_{p}+R_{p}\theta_{p}\sin\varphi -x_{g}-R_{g}\theta_{g}\sin\varphi \\ \delta_{y}=y_{p}+R_{p}\theta_{p}\cos\varphi -y_{g}-R_{g}\theta_{g}\cos\varphi \end{array}\\ \delta_{z}=z_{p}-z_{g}\\ \delta_{n}=(\delta_{x}\sin\varphi +\delta_{y}cos\varphi )\cos\beta_{g}+\delta_{z}\sin\beta_{g}\\ {\dot{\delta }}_{n}=({\dot{\delta }}_{x}\sin\varphi +{\dot{\delta }}_{y}cos\varphi )\cos\beta_{g}+{\dot{\delta }}_{z}\sin\beta_{g} \end{array}$$

Due to the nonlinearity of the clearance, the dynamic meshing force can be expressed:

(46) $$\{\begin{array}{r} \begin{array}{cc} F_{n}=k_{m}(\delta_{n}-b-e)+c_{m}({\dot{\delta }}_{n}-\dot{e}) & (\delta_{n}-e)/b>1 \end{array}\\ \begin{array}{cc} 0 & -1\leq (\delta_{n}-e)/b\leq 1 \end{array}\\ \begin{array}{cc} F_{n}=k_{m}(\delta_{n}+b-e)+c_{m}({\dot{\delta }}_{n}-\dot{e}) & (\delta_{n}-e)/b<-1 \end{array} \end{array}$$

where, *b* is the clearance and *e* is the comprehensive meshing error. Then the following non-dimensional equations are conducted by the nonlinear dynamic Equations (43) and (44) [46]:

(47) $$\{\begin{array}{l} \begin{array}{l} m_{p}b\omega_{n}^{2}{\ddot{\bar{x}}}_{p}+c_{bpy}b\omega_{n}{\dot{\bar{x}}}_{p}+k_{bpy}b{\bar{x}}_{b}+{\bar{F}}_{n}\cos\beta_{g}\sin\varphi =0\\ m_{p}b\omega_{n}^{2}{\ddot{\bar{y}}}_{p}+c_{bpy}b\omega_{n}{\dot{\bar{y}}}_{p}+k_{bpy}b{\bar{y}}_{b}+{\bar{F}}_{n}\cos\beta_{g}\cos\varphi =0 \end{array}\\ m_{p}b\omega_{n}^{2}{\ddot{\bar{z}}}_{p}+c_{bpy}b\omega_{n}{\dot{\bar{z}}}_{p}+k_{bpy}b{\bar{z}}_{p}+{\bar{F}}_{n}\sin\beta_{g}=0\\ I_{p}b\omega_{n}^{2}{\ddot{\bar{\theta }}}_{p}R_{bp}-t_{p}R_{bp}+{\bar{F}}_{n}R_{bp}^{2}\cos\beta_{g}=0 \end{array}$$

(48) $$\{\begin{array}{l} \begin{array}{l} m_{g}b\omega_{n}^{2}{\ddot{\bar{x}}}_{g}+c_{bgy}b\omega_{n}{\dot{\bar{x}}}_{g}+k_{bgy}b{\bar{x}}_{g}-{\bar{F}}_{n}\cos\beta_{g}\sin\varphi =0\\ m_{g}b\omega_{n}^{2}{\ddot{\bar{y}}}_{g}+c_{bgy}b\omega_{n}{\dot{\bar{y}}}_{g}+k_{bgy}b{\bar{y}}_{g}-{\bar{F}}_{n}\cos\beta_{g}\cos\varphi =0 \end{array}\\ m_{g}b\omega_{n}^{2}{\ddot{\bar{z}}}_{g}+c_{bgy}b\omega_{n}{\dot{\bar{z}}}_{g}+k_{bgy}b{\bar{z}}_{g}-{\bar{F}}_{n}\sin\beta_{g}=0\\ I_{g}b\omega_{n}^{2}{\ddot{\bar{\theta }}}_{g}R_{bg}+t_{g}R_{bg}-{\bar{F}}_{n}R_{bg}^{2}\cos\beta_{g}=0 \end{array}$$

The generalized coordinates of the system can be defined as:

(49) $$x=[\begin{array}{ccccc} \begin{array}{cc} {\bar{x}}_{p} & {\bar{y}}_{p} \end{array} & {\bar{z}}_{p} & \begin{array}{cc} {\bar{x}}_{g} & {\bar{y}}_{g} \end{array} & {\bar{z}}_{g} & {\bar{\theta }}_{p}R_{bp}-{\bar{\theta }}_{g}R_{bg} \end{array}]$$

The variable *x* in the generalized coordinates of the system is substituted into Equation (46), and the dynamic equation of the system can be denoted as:

(50) $$\{\begin{array}{l} {\ddot{x}}_{1}+\frac{c_{bpx}}{m_{p}\omega_{p}}{\dot{x}}_{1}+\frac{k_{bpx}}{m_{p}\omega_{n}^{2}}x_{1}+\frac{{\bar{F}}_{n}\cos\beta_{g}\sin\varphi }{m_{p}b\omega_{n}^{2}}=0\\ {\ddot{x}}_{2}+\frac{c_{bpy}}{m_{p}\omega_{p}}{\dot{x}}_{2}+\frac{k_{bpy}}{m_{p}\omega_{n}^{2}}x_{2}+\frac{{\bar{F}}_{n}\cos\beta_{g}\cos\varphi }{m_{p}b\omega_{n}^{2}}=0\\ {\ddot{x}}_{3}+\frac{c_{bpz}}{m_{p}\omega_{p}}{\dot{x}}_{3}+\frac{k_{bpz}}{m_{p}\omega_{n}^{2}}x_{3}+\frac{{\bar{F}}_{n}\sin\beta_{g}}{m_{p}b\omega_{n}^{2}}=0\\ {\ddot{x}}_{4}+\frac{c_{bgx}}{m_{g}\omega_{g}}{\dot{x}}_{4}+\frac{k_{bgx}}{m_{g}\omega_{n}^{2}}x_{4}-\frac{{\bar{F}}_{n}\cos\beta_{g}\sin\varphi }{m_{g}b\omega_{n}^{2}}=0\\ {\ddot{x}}_{5}+\frac{c_{bgy}}{m_{p}\omega_{p}}{\dot{x}}_{5}+\frac{k_{bgy}}{m_{p}\omega_{n}^{2}}x_{5}-\frac{{\bar{F}}_{n}\cos\beta_{g}\cos\varphi }{m_{p}b\omega_{n}^{2}}=0\\ {\ddot{x}}_{6}+\frac{c_{bgz}}{m_{g}\omega_{n}}{\dot{x}}_{6}+\frac{k_{bgz}}{m_{g}\omega_{n}^{2}}x_{6}-\frac{{\bar{F}}_{n}\sin\beta_{g}}{m_{g}b\omega_{n}^{2}}=0\\ {\ddot{x}}_{7}=\frac{1}{b\omega_{n}^{2}}\left[\left(\frac{t_{p}R_{bp}}{I_{p}}+\frac{t_{g}R_{bg}}{I_{g}}\right)-\left(\frac{{\bar{F}}_{n}\cos\beta_{g}\cos\varphi R_{bg}^{2}}{I_{p}}+\frac{{\bar{F}}_{n}\cos\beta_{g}\cos\varphi R_{bg}^{2}}{I_{g}}\right)\right] \end{array}$$

### 3.3. Analysis of Nonlinear Dynamic Characteristics of the Helical Gear Transmission System

**Figure 13:**
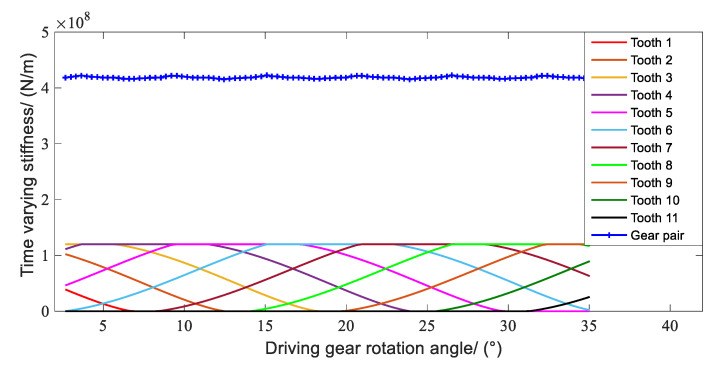
Time varying meshing stiffness of the first gear pair.

The parameters of a helical gear system are shown in Table 3. The time-varying meshing stiffness (TVMS) of the gear pair was calculated by the numerical analysis method [47], and the results are shown in Figure 13. In this Figure, with the gear rotation, the teeth approach and recess in turn as from Gear 1 to Gear 11. Additionally, there are multiple curves that coincidence at the same time, this is because there is more than one tooth engaged in meshing during the gear meshing process simultaneously. According to the parallel relation between the simultaneous meshing teeth, the TVMS of the gear pair can be obtained.

In actual working conditions, the bearing has six degrees of freedom to move and rotate along *x*, *y*, and *z* directions. In this case, the bearing stiffness is calculated by:

(51) $$K_{b}=\left[\begin{array}{llllll} k_{xx} & k_{xy} & k_{xz} & k_{x\theta_{x}} & k_{x\theta_{y}} & k_{x\theta_{z}}\\ k_{yx} & k_{yy} & k_{yz} & k_{y\theta_{x}} & k_{y\theta_{y}} & k_{y\theta_{z}}\\ k_{zx} & k_{zy} & k_{zx} & k_{z\theta_{x}} & k_{z\theta_{y}} & k_{z\theta_{z}}\\ k_{\theta_{x}x} & k_{\theta_{x}y} & k_{\theta_{x}z} & k_{\theta_{x}\theta_{x}} & k_{\theta_{x}\theta_{y}} & k_{\theta_{x}\theta_{z}}\\ k_{\theta_{y}x} & k_{\theta_{y}y} & k_{\theta_{y}z} & k_{\theta_{y}\theta_{x}} & k_{\theta_{y}\theta_{y}} & k_{\theta_{y}\theta_{z}}\\ k_{\theta_{z}x} & k_{\theta_{z}y} & k_{\theta_{z}z} & k_{\theta_{z}\theta_{x}} & k_{\theta_{z}\theta_{y}} & k_{\theta_{z}\theta_{z}} \end{array}\right]$$

While, the gear dynamic model established in this paper only considers the movement along *x*, *y*, and *z* directions and the rotation around *z* direction, according to the axial and radial stiffness calculated by Equations (39) and (41), the bearing stiffness in the dynamic model can be established as:

(52) $$K_{b}=\left[\begin{array}{llll} k_{bpx} & 0 & 0 & 0\\ 0 & k_{bpy} & 0 & 0\\ 0 & 0 & k_{bpz} & 0\\ 0 & 0 & 0 & 0 \end{array}\right]=\left[\begin{array}{llll} k_{xx} & 0 & 0 & 0\\ 0 & k_{yy} & 0 & 0\\ 0 & 0 & k_{zx} & 0\\ 0 & 0 & 0 & 0 \end{array}\right]=1000\times \left[\begin{array}{llll} K_{r} & 0 & 0 & 0\\ 0 & K_{r} & 0 & 0\\ 0 & 0 & K_{a} & 0\\ 0 & 0 & 0 & 0 \end{array}\right]$$

**Figure 14:**
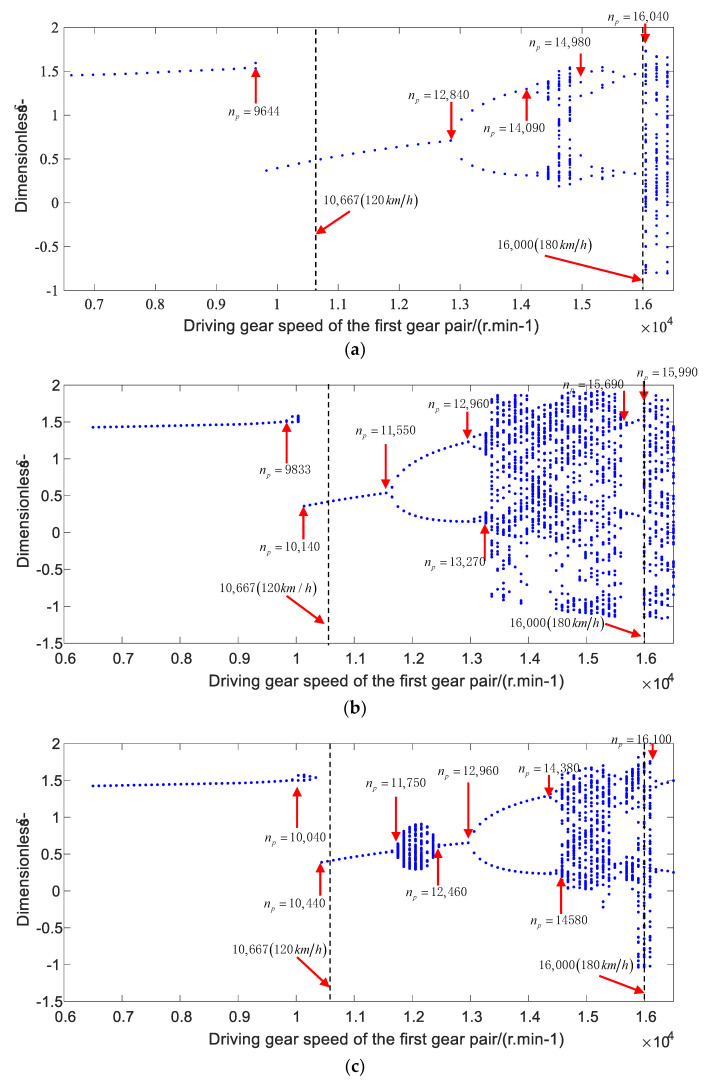
The bifurcated diagrams of gear system for the accelerating working condition: (**a**) constant bearing stiffness, (**b**) variable bearing stiffness values without considering the influence of thermal deformation, and (**c**) variable bearing stiffness values under the influence of thermal deformation.

**Figure 15:**
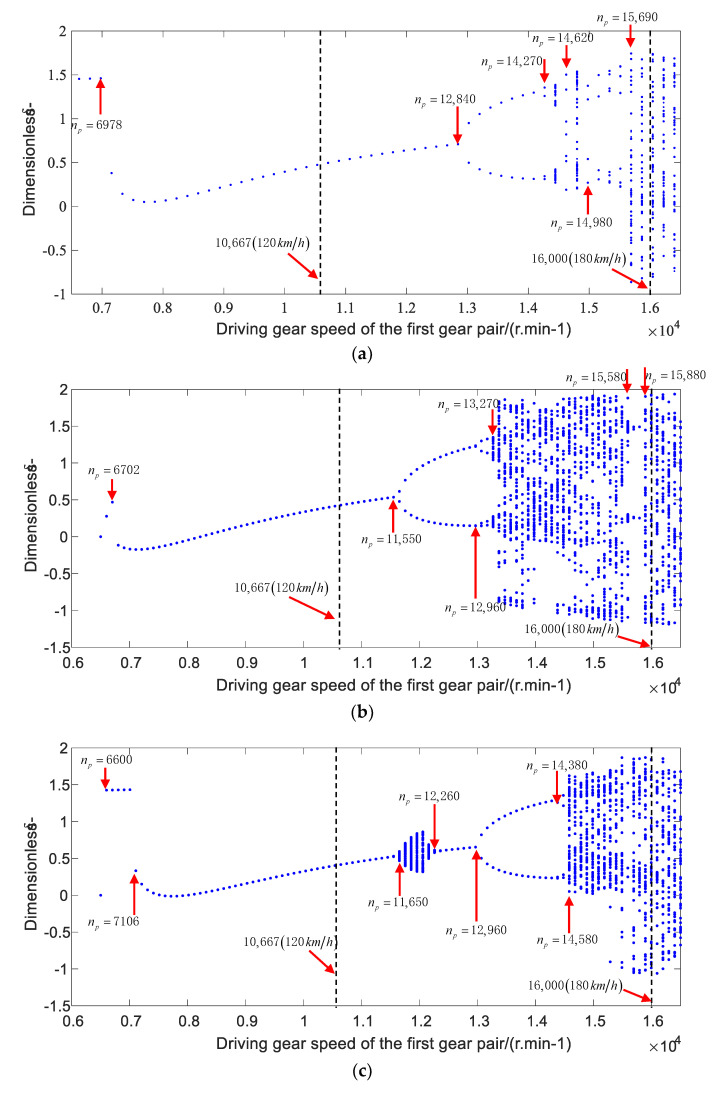
The bifurcated diagrams of gear system for the decelerating working condition: (**a**) constant bearing stiffness, (**b**) variable bearing stiffness values without considering the influence of thermal deformation, and (**c**) variable bearing stiffness values considering the influence of thermal deformation.

Thus, the bearing stiffness and time varying meshing stiffness of gear pair were substituted into Equation (48), and the Runge–Kutta method [46] was used to obtain the approximate solution of the nonlinear equation of the gear-bearing system. The nonlinear dynamic characteristics of the gear system under the conditions of acceleration and deceleration were calculated, respectively, by using the gear parameters in Table 3. The bifurcated diagrams of the dynamic transmission error under accelerating and decelerating working conditions are shown in Figure 14 and Figure 15, respectively.

Figure 14a–c shows the bifurcated diagrams of dynamic transfer error of gear pair with the constant bearing stiffness, variable bearing stiffness values without the thermal deformation, and variable bearing stiffness values with the thermal deformation, respectively, under the accelerating condition. The results show that the system moves in a periodic-one motion at a low speed, and occurs the periodic motions in a range of 9644 r/min (with the constant bearing stiffness), 9933−10,140 r/min (without considering the thermal deformation), and 10,040−10,440 r/min (considering the thermal deformation). Then, the step phenomenon occurs at the rotating speed of 6978 r/min (with the constant bearing stiffness), 10,140 r/min (without considering the thermal deformation), and 10,440 r/min (considering the thermal deformation). After that, the system performs a periodic-one motion, it exhibits a chaotic motion in the range of 11,750−12,460 r/min, and proceeds the 2T-priodicic motion at 12,840 r/min (with the constant bearing stiffness), 11,550 r/min (without considering the thermal deformation), and 12,960 r/min (considering the thermal deformation), respectively. Moreover, the system undergoes a chaotic motion in the rotating speed range of 14,950−16,040 r/min (with the constant stiffness), 12,960−13,270 r/min and 15,690−15,990 r/min (without considering the thermal deformation), 14,380−14,580 r/min and 16,100−16,500 r/min (considering the thermal deformation), and it proceeds into a chaotic motion during other speed ranges.

Under the decelerating condition with the rotating speed range of 16,500−6500 r/min, the bifurcated diagrams of dynamic transfer error of gear pairs with the constant bearing stiffness, variable bearing stiffness values without considering the influence of thermal deformation, and variable bearing stiffness values considering the thermal deformation are shown in Figure 15a−c, respectively. The results are similar to those in the accelerating condition that the nonlinear bifurcation phenomena, such as the 1T-priodicic motion, the quasi-periodic motion and the chaos motion, also exist in the gear system with the increase of rotating speed. The system exhibited a spike at the speed of 6978, 6702, 6600, and 7106 r/min, respectively, and it exhibits a 2T-priodicic motion at the speed of 12,840 r/min (with the constant bearing stiffness), 11,550 r/min (without considering the thermal deformation), and 12,960 r/min (considering the thermal deformation), respectively. In addition, the system performs a periodic motion in the rotating speed range of 14,270−14,620 r/min and 14,990−15,690 r/min (with the constant bearing stiffness), 12,960−13,270 r/min and 15,580−15,880 r/min (without considering the thermal deformation), and 14,380−14,590 r/min (considering the thermal deformation), and the system proceeds to chaotic behaviors during other speed ranges. Compared with the dynamic response of the system during the accelerating condition, the difference is that the hopping phenomenon of these three conditions occurs below 7500 r/min, and the dynamic transfer error in the low speed range is less than that under the accelerating condition. When considering the bearing thermal deformation, the dynamic response of the system in the high speed range is a periodic motion under the accelerating condition, which is different from the chaotic motion under the decelerating condition.

Through the comparison of the bifurcated diagrams of the transmission error under the accelerating and decelerating conditions, it can be seen that the bearing temperature rise has a great impact on the nonlinear dynamic characteristics of the system within the range of medium and high speeds. When the considering the bearing temperature rise, the system will have a chaotic motion around the speed of 12,000 r/min. By comparing the nonlinear dynamic characteristics of the gear system under the decelerating and accelerating conditions, the nonlinear behaviors of the system in the medium speed range are similar under three different bearing stiffness values. However, the bifurcation characteristics of the system are different in the low or high speed range. The spike phenomenon may easily occur in the low speed range under the decelerating condition, while the system will change from the chaotic motion to the periodic motion in the high speed range (16,100−16,500 r/min) under the accelerating condition, when considering the influence of thermal deformation.

## 4. Conclusions

The thermal deformation calculation model of the deep groove ball bearing was established through the statics and pseudo-statics analysis in this study. The heat generation and temperature rise of the deep groove ball bearing were analyzed under the high speed working condition. The deformation, radial and axial stiffness of bearing under the temperature rise were also calculated based on the Newton–Raphson method. The dynamic model of helical gear transmission system was established. Considering the thermal deformation of the bearing, the influence of the various bearing stiffness values on nonlinear dynamic characteristics of gear system was studied. The following specific conclusions can be drawn:

(1) Under the condition of high speed working condition, the thermal deformation of the bearing will occur in both the axial and radial directions. The axial thermal deformation is far less than the axial deformation, but the radial thermal deformation is close to the bearing radial deformation under loading. When considering the thermal deformation of the bearing, the axial stiffness of the bearing is reduced, while the radial stiffness increases.
(2) The gear system of high speed electric drive appears T-periodic and chaotic motions under both accelerating and decelerating conditions. Under the accelerating condition, the system has a hopping point around 10,000 rpm, and it exhibits the 2T-periodic motion without considering the thermal deformation, while the rotational speed range of the system with the 2T-periodic motion is large when considering the thermal deformation. The system will have a spike step before the rotating speed of 7000 rpm under the decelerating condition, and there is one hopping point under the bearing stiffness without considering the thermal deformation. On the contrary, the system has two spikes under the bearing stiffness considering the influence of thermal deformation.
(3) In accelerating and decelerating conditions, the bifurcation behavior of the system with the constant bearing stiffness is better than that with variable bearing stiffness values within the range of medium and high speed. The bifurcation characteristics of the system without considering the thermal deformation are more complicated than that considering the influences of the thermal deformation, but the extra chaotic motions of the system will appear when considering the thermal deformation in the medium speed range (about 11,150−11,250 r/min), which shows that the bearing stiffness may change the nonlinear dynamic characteristics of the system. Therefore, the influence of thermal deformation on bearing stiffness should be considered in the dynamic analysis of the high speed electric drive helical gear system.

## Figures and Tables

**Table 1:** Parameters of the deep groove ball bearing.

Parameters	Deep Groove Ball Bearing
Number of balls	9
Bearing ball diameter (mm)	12
Bearing bore diameter (mm)	40
Bearing outside diameter (mm)	80
Radius of curvature of inner channel (mm)	6.24
Radius of curvature of outer channel (mm)	6.36
Coefficient of linear expansion	1.36 × 10^−7^
Initial contact angle (°)	14.8351
The initial clearance (mm)	0.04
Bearing pitch diameter (mm)	60

**Table 2:** Thermal resistance description table.

Name of the Node	Equations of the Thermal Resistance	Name of the Node	Equations of The Thermal Resistance
*R_ci-s_*	$R_{ci-s}=\frac{1}{k_{s}\pi B}+\frac{4L_{s}}{k_{s}\pi D_{s}^{2}}+\frac{1}{h_{s}\pi D_{s}^{2}}$	*R_h-a-ax_*	$R_{h-a-ax}=\frac{4L_{h}}{k_{h}\pi \left(D_{h}^{2}-D_{o}^{2}\right)}+\frac{4L_{h}}{k_{h}\pi D_{o}^{2}}+\frac{4}{h_{h}\pi D_{h}^{2}}$
*R_i-ci_*	$R_{i-ci}=\frac{\ln\left(D_{i}/D_{s}\right)}{2k_{i}\pi B}$	*R_s_*_-*og*_	$R_{s-og}=\frac{Z}{h_{s}\pi D_{s}^{2}}$
*R_b-i_*	$R_{b-i}=\frac{1}{k_{b}\pi D_{b}}$	*R_i_*_-*og*_	$R_{i-og}=\frac{1}{h_{i}\pi D_{i}B}$
*R_b-o_*	$R_{b-o}=\frac{1}{k_{b}\pi D_{b}}$	*R_b_*_-*og*_	$R_{b-og}=\frac{1}{h_{b}\pi D_{b}^{2}}$
*R_o-co_*	$R_{o-co}=\frac{\ln\left(D/D_{o}\right)}{2k_{o}\pi C}$	*R_o_*_-*og*_	$R_{o-og}=\frac{1}{h_{o}\pi D_{o}C}$
*R_co-h_*	$R_{co-h}=\frac{\ln\left(D_{h}/D_{o}\right)}{2h_{h}\pi C}+\frac{1}{k_{h}\pi D_{h}L_{{}_{h}}}$	*R_h-a_*	$R_{h-a}=\frac{R_{h-a-ax}R_{h-a-r}}{R_{h-a-ax}+R_{h-a-r}}$
*R_h-a-r_*	$R_{h-a-r}=\frac{\ln\left(D_{h}/D_{o}\right)}{2k_{h}\pi C}+\frac{1}{h_{h}\pi D_{h}L_{h}}$		

**Table 3:** First gear pair parameters of the high speed electric drive powertrain system.

Parameters	Driving Gear	Driven Gear
Number of teeth	29	78
Normal module (mm)	1.537	
Pressure angle (°)	16.5	
Face width (mm)	33	
Helix angle (°)	31.2	
Elastic modulus (MPa)	20,600	
Material density (tonne.mm-3)	7.8×10^9^	
Mass (kg)	1.201	3.1
Poisson ratio	0.25	
Rotational inertia (tonne.mm^2^)	0.1152	1.6

## Data Availability

All data generated or appeared in this study are available upon request by contact with the corresponding author. Furthermore, the models and code used during the study cannot be shared at this time as the data also forms part of an ongoing study.

## Nomenclature

Γ	Complete elliptic integral of the first kind
Π	Complete elliptic integral of the second kind
*κ*	Ellipticity parameter
*α*_0_	Free contact angle of bearing (rad)
*α*	mounted contact angle (rad)
*δ*	Relative displacement or elastic deformation of contact (mm)
*δ_n_*	Contact deformation between teeth (m)
*θ*	The angle of bearing under the action of external force (rad)
*ψ*	Position angle of ball (rad)
*v*	Poisson’s ratio
*E_s_*	Modulus of elasticity (Mpa)
*ρ_b_*	Density of ball (kg/mm^3^)
*ρ*	Density of gear (kg/m^3^)
*ω_c_*	Rotational speed refers to orbital motion (rad/s)
*μ*	Lubricant kinematic viscosity (centistokes)
*μ_s_*	Friction coefficient between ball and raceway
*ω*	The relative angular speed (rad/s)
*ω_si_*	Spinning motion of ball relative to the outer raceway (rad/s)
*ω_so_*	Spinning motion of ball relative to the outer raceway (rad/s)
*ω_n_*	The absolute angular speed of the inner raceway (rad/s)
*ω_b_*	Spinning speed of ball (rad/s)
*β*	Attitude angle (rad)
*β_g_*	Gear helix angle (rad)
*φ*	The angle between the acting surface and vertical direction (rad)
*θ_p_*	Rotation angle of driving gear (rad)
*θ_g_*	Rotation angle of driven gear (rad)
*A*_1_	The axial distance between the loci of inner and outer raceway groove curvature center (mm)
*A*_2_	The radial distance between the loci of outer and outer raceway groove curvature center (mm)
*A*	Semimajor axis of projected contact ellipse (mm)
*a*	Thermal coefficient of expansion (1/°C)
*B*	Width of the inner ring (mm)
*B_f_*	Total curvature
*b*	Backlash (m)
*C*	Width of the outer ring (mm)
*c_m_*	Gear meshing damping (N.s/m)
*c_b_*	Bearing damping coefficients (N.s/m)
*D_h_*	The distance of the bearing seat from the center line of the shaft (mm)
*D_m_*	Pitch diameter of bearing (mm)
*D*	Diameter (mm)
*d*	Raceway diameter (mm)
*e*	Integrated meshing error of gear pair (mm)
*f_i_*	Curvature coefficient
*f*_0_	A factor depending on the type of bearing and the method of lubrication
*F*	Applied force on bearing (N)
*F_c_*	Centrifugal force (N)
*F_n_*	Meshing force of gear pair (N)
*H*	Power (W)
*h*	Convective heat transfer coefficient (W/mm^2^/°C)
I	Moment of inertia (kg.m^2^)
*J_b_*	Mass moment of inertia of ball (kg.mm^2^)
*K*	Load-deflection constant (N/mm^1.5^)
*k*	Thermal conductivity (W/mm/°C)
*k_v_*	Thermal conductivity of lubrication oil (W/mm/°C)
*K_a_*	Axial stiffness of bearing (N/mm)
*K_r_*	Radial stiffness of bearing (N/mm)
*k_bpx_*	Bearing stiffness of bearing used to support driving gear (N/m)
*k_bgx_*	Bearing stiffness of bearing used to support driven gear (N/m)
*k_m_*	Meshing stiffness of gear pair (N.m)
*L_s_*	Distance between the node *T_s_* from the transmission shaft end (mm)
*L_h_*	Height of bearing housing (mm)
*M_g_*	Gyroscopic moment (N.mm)
*M_μ_*	Bearing friction torque due to lubrication (N.mm)
*M*_1_	Bearing friction torque due to load (N.mm)
*M_s_*	Spinning friction moment (N.mm)
*m*	Quality (kg)
*n*	Rotation speed of bearing (r/min)
*n_v_*	One third of the speed of the cage (m/s)
*P_γ_*	Prandtl number
*Q*	Ball normal load (N)
*Q_r_*	Radial direction load on ball (N)
*Q_a_*	Axial direction load on ball (N)
*R_r_*	Radius to locus of raceway groove curvature centers (mm)
*R_b_*	Gear base radius (m)
*R*	Thermal resistant between nodes (°C/W)
*r*	Radius of curvature (mm)
*T*	Temperature of node (°C)
*T_a_*	Temperature of air (°C)
*t*	Torque applied on gear (Nm)
*u_T_*	Radial thermal deformation (mm)
*u_Ti’_*	Radial thermal deformation of inner ring (mm)
*u_To’_*	Radial thermal deformation of outer ring (mm)
*u_Tri_*	Radial thermal deformation of inner raceway considering thermal deformation of shaft (mm)
*u_Tro_*	Radial thermal deformation of outer raceway considering thermal deformation of bearing seat (mm)
*u_Tr_*	The relative radial thermal deformation between inner and outer raceway considering thermal deformation of ball (mm)
*u_a_*	The relative axial thermal deformation between the inner and outer raceway (mm)
*u_all_*	Composite deformation (mm)
*X*_1_	Axial projection of distance between ball center and outer raceway groove curvature center (mm)
*X*_2_	Radial projection of distance between ball center and outer raceway groove curvature center (mm)
*Z_b_*	Number of balls
**Subscripts**	
*a*	Axial direction
*r*	Radial direction
*b*	Ball
*n*	Normal direction
*i*	Inner ring or raceway
*o*	Outer ring or raceway
*ci*	Contact point between inner ring and transmission shaft
*co*	Contact point between outer ring and bearing seat
*j*	The *j*_th_
*s*	Transmission shaft
*h*	Bearing seat
*p*	Driving gear
*g*	Driven gear
*x*	Refers to x direction
*y*	Refers to y direction
*z*	Refers to z direction
*og*	gas–oil
**Abbreviations**	
NVH	noise: vibration, and harshness
TVMS	The time-varying meshing stiffness
